# Targeted HIV testing at birth supported by low and predictable mother‐to‐child transmission risk in Botswana

**DOI:** 10.1002/jia2.25111

**Published:** 2018-05-29

**Authors:** Maryanne Ibrahim, Kenneth Maswabi, Gbolahan Ajibola, Sikhulile Moyo, Michael D Hughes, Oganne Batlang, Maureen Sakoi, Chloe Auletta‐Young, Laura Vaughan, Shahin Lockman, Patrick Jean‐Philippe, Xu Yu, Matthias Lichterfeld, Daniel R Kuritzkes, Joseph Makhema, Roger L Shapiro

**Affiliations:** ^1^ Harvard Medical School Doris Duke International Clinical Research Fellowship Boston MA USA; ^2^ University of California Los Angeles David Geffen School of Medicine Los Angeles CA USA; ^3^ Botswana Harvard AIDS Institute Partnership Gaborone Botswana; ^4^ Department of Biostatistics Harvard T.H Chan School of Public Health Boston MA USA; ^5^ Department of Immunology and Infectious Diseases Harvard T.H Chan School of Public Health Boston MA USA; ^6^ Infectious Disease Division Brigham and Women's Hospital Boston MA USA; ^7^ National Institute of Allergy and Infectious Diseases National Institutes of Health Bethesda MD USA; ^8^ Infectious Disease Division Massachusetts General Hospital Boston MA USA

**Keywords:** HIV, mother‐to‐child transmission, children, paediatrics, vertical transmission, viral suppression

## Abstract

**Introduction:**

Most African countries perform infant HIV testing at 6 weeks or later. The addition of targeted testing at birth may improve retention in care, treatment outcomes and survival for HIV‐infected infants.

**Methods:**

HIV‐exposed infants were screened as part of the Early Infant Treatment (EIT) study in Botswana. Screened infants were ≥35 weeks gestational age and ≥2000 g at birth. Risk factors for mother‐to‐child transmission (MTCT) were assessed by maternal obstetric card or verbally. Risk factors included <8 weeks ART in pregnancy, last known CD4 <250 cells/mm^3^, last known HIV RNA >400 copies/mL, poor maternal ART adherence, lack of maternal zidovudine (ZDV) in labour, or lack of infant post‐exposure prophylaxis. Infants underwent dried blood spot testing by Roche Cobas Ampliprep/Cobas Taqman HIV‐1 qualitative PCR.

**Results:**

From April 2015 to April 2016, 2303 HIV‐exposed infants were tested for HIV in the EIT study. Of these, 369 (16%) were identified as high risk for HIV infection by information available at birth, and 12 (0.5% overall, 3.25% of high risk) were identified as HIV positive at birth. All 12 positive infants were identified as high risk at the time of screening, and only 2 risk factors were required to identify all positive infants: either <8 weeks of maternal ART in pregnancy (75%) or lack of maternal HIV suppression at last test (25%).

**Conclusions:**

*In utero* MTCT occurred only among infants identified as high risk at delivery, using information available from the mother or obstetric record. Birth testing that targets high‐risk infants based on maternal ART receipt is likely to identify the majority of *in utero* HIV transmissions, and allows early ART initiation for these infants.

## 1 Introduction

The World Health Organization (WHO) recommends HIV testing for all HIV‐exposed infants at 4 to 6 weeks of age 1, based on studies demonstrating the benefits of early infant HIV treatment 2, 3. While 4 to 6 week testing allows sufficient time to detect both *in utero* and intrapartum mother‐to‐child HIV transmission (MTCT), limitations include early infant mortality prior to diagnosis, children lost to follow‐up, and seeding of cells comprising the long‐lived viral reservoir prior to treatment initiation 3, 4, 5. In Botswana, the Ministry of Health reported a 1.5% MTCT rate in 2015, utilizing a 6‐week testing strategy 6. However, only 68% of HIV‐exposed infants were tested within the recommended period, and when testing occurred, a substantial number of mothers did not receive results for their children 6. In recognition of similar challenges throughout the developing world 7, WHO released a conditional recommendation in June 2016 that countries consider the addition of nucleic acid testing at birth to existing diagnostic testing approaches to improve early initiation of treatment and reduce loss to follow‐up of HIV‐infected infants 8.

South Africa has now transitioned to a model of universal birth testing and 10‐week testing to identify HIV‐infected infants 9. This two‐test model allows for earlier detection of *in utero* HIV transmission while continuing to capture intrapartum transmission, but it doubles the cost of early infant testing. We sought to determine whether a more targeted approach to birth testing is possible with a more efficient and lower cost strategy.

In 2015, the Botswana‐Harvard Partnership launched the Early Infant Treatment (EIT) Study. As part of this study, HIV‐exposed infants are tested at birth and, if HIV positive, offered immediate antiretroviral therapy. This manuscript summarizes the first year of early infant testing in the EIT study, evaluating specific maternal risk factors identifiable at delivery that may contribute to MTCT and allow for targeted birth testing of infants.

## 2 Methods

### 2.1 Trial design and study population

Between April 2015 and April 2016, HIV‐exposed infants <96 hours of age were screened for HIV in the Gaborone and Francistown regions of Botswana as part of the Botswana‐Harvard Partnership EIT Study. Screening was conducted at five hospital maternity wards (Princess Marina Hospital in Gaborone, Scottish Livingstone Hospital in Molepolole, Deborah Reteif Memorial Hospital in Mochudi, Nyangabgwe Referral Hospital in Francistown, and Selebi Phikwe Government Hospital in Selebi Phikwe), and in surrounding maternity clinics. There was staggered opening of screening at the different sites: Gaborone sites opened screening in April 2015, and all other sites were opened by September 2015.

Infants were screened based on the following inclusion criteria: mother/guardian ≥18 years of age and able to provide informed consent, gestational age at birth ≥35 weeks, birth weight ≥2000 g , age <96 hours after birth, ability to initiate antiretroviral therapy (ART) within 7 days after birth, and eligible for ART through the Botswana government programme (which provides ART for pregnant or breastfeeding HIV‐infected citizens irrespective of CD4 cell count). Infants were excluded from screening if they were hospitalized for severe medical illness or if they had a medical condition making it unlikely that the infant would survive.

All mothers of screened infants signed written consent approved by ethical review boards in Botswana (Health Research Development Committee) and Boston (Harvard T.H. Chan School of Public Health Office of Human Research Administration).

### 2.2 Study procedures and monitoring

Screening for infant HIV infection was conducted by trained research assistants, who asked HIV‐infected women on maternity wards (either pre‐ or post‐delivery) if they agreed to have their infant HIV‐tested. A recruitment script was followed that described the available study for those infants found to be HIV infected, with appropriate counselling given by the research assistants. Interested mothers were initially asked to provide informed consent for screening only, and all mothers were assessed for MTCT risk factors by their obstetric card and/or verbally to identify those at high risk for transmission. Risk factors included <8 weeks ART in pregnancy, last known CD4 to be <250 cells/mm^3^ (not necessarily during current pregnancy), last known HIV RNA to be above the threshold for detection in Botswana of >400 copies/mL (not necessarily during current pregnancy), poor maternal ART adherence (defined as one or more missed doses of ART during pregnancy, including stopping ART completely), lack of maternal zidovudine (ZDV) in labour, or lack of infant post‐exposure prophylaxis 10. When an MTCT risk factor was identified, women were also offered a second HIV PCR test for their infant at 10 to 14 days, in addition to the initial testing. All women were also reminded to have their infant return at 6 weeks for routine HIV PCR testing at a government facility.

Following maternal consent, infants had a heel stick and 3 to 5 dried blood spots (DBS) collected. DBS samples were tested for HIV PCR utilizing Roche Cobas Ampliprep/Cobas Taqman HIV‐1 Qualitative Polymerase Chain Reaction (PCR) testing (Roche Diagnostics, Mannheim, Germany) at the Botswana‐Harvard HIV Reference Laboratory (BHHRL), adjacent to Princess Marina Hospital in Gaborone 10. Turn‐around time for this DNA PCR testing was 1 day in most cases, and was made available to mothers at the hospital or at the study clinic. After appropriate post‐test counselling, HIV‐infected infants were offered enrolment in the longitudinal EIT Study, and immediate ART initiation occurred following a second consent process.

After enrolment in the longitudinal EIT Study, mothers of HIV‐infected infants were tested to evaluate their CD4 cell count and HIV RNA levels at BHHRL using whole blood on BD FACS Calibur Flow Cytometer (Becton Dickinson Biosciences, Belgium) and Beckman Coulter Abbott m2000sp/m2000rt (Abbott Molecular Inc, Des Plaines, IL, USA) respectively.

### 2.3 Data management and statistical analysis

Data from maternities, including the number of HIV‐positive women delivering, number discharged and number eligible for screening, were stored in Microsoft Excel. Data for screened EIT Study participants were collected using REDCap data capture tools hosted at Harvard T.H. Chan School of Public Health 11. These data included documentation of MTCT risk factors and results of all laboratory testing. All results are presented as descriptive statistics.

## 3 Results

**Figure 1:**
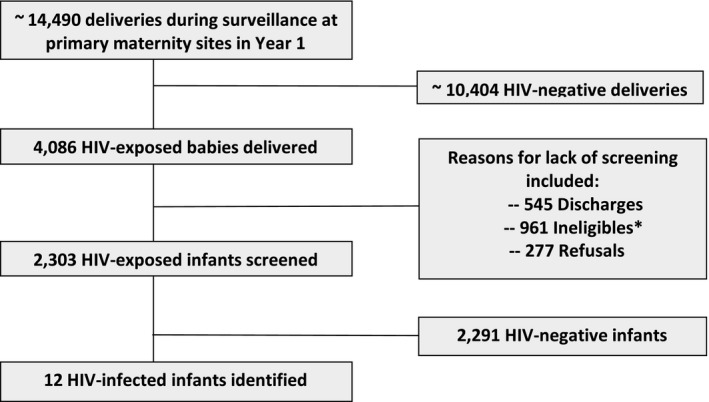
Screening consort diagram. *Ineligibles included Gestational Age <35 weeks, birth weight <2500 g, non‐citizens, unfit to consent, stillbirth, babies in Neonatal Unit.

In the first year of the EIT Study, approximately 14,490 infants were delivered in total during the screening periods for each maternity site. Of these, 4086 HIV‐exposed infants were delivered, translating to an estimated 28.2% HIV prevalence among women delivering at these maternity sites. Of these 4086 infants, 3541 (87%) had not been discharged, 2,580 (63%) were eligible for screening according to EIT study criteria, and 2303 (56%) ultimately agreed to be screened for HIV (Figure 1). Reasons for screening refusal (11% of the eligible mothers) included a desire to consult their partner, a wish to test only at 6 weeks according to Botswana government guidelines, enrolment in another study, or fear of the stigma of HIV.

Of the 2303 infants screened, all of whom were assessed for high‐risk factors via their obstetric card and/or verbally, 369 (16%) were identified as high risk for one or more of the following reasons: less than 8 weeks maternal ART in pregnancy (43%), maternal CD4 cell count known to be <250 cells/mm^3^ at last test (19%), HIV RNA known to be >400 copies/mL at last test (2%), poor ART adherence reported in pregnancy (4%), lack of infant post‐exposure prophylaxis (3%), no maternal zidovudine during labour (22%), and other/unspecified reasons (17%) (Table 1). Receiving <8 weeks of ART in pregnancy was documented for 157 women, or nearly 7% of all HIV‐positive women who delivered at the maternity sites and had an infant eligible for screening.

**Table 1:** *In utero* MTCT by identified risk factors at birth

	HIV‐positive infants with risk factor/Number screened with risk factor^a^
Any risk factor	12/369 (3·25%)
Less than 8 weeks maternal ART in pregnancy	9/157
Maternal CD4 known to be <250 at last test	1^b^/69
HIV RNA known to be >400 copies/mL at last test	3/6
Poor ART adherence reported in pregnancy	1^b^/16
Lack of infant post‐exposure prophylaxis	0/12
No maternal zidovudine during labour	0/81
Other^c^	0/30
Unspecified	0/32

a 403 total risk factors were identified among 369 infants with at least one risk factor.

b Reported in addition to <8 weeks of maternal ART in pregnancy.

c Other included last known CD4 between 250 and 350 cells/mm^3^, birth before arrival at hospital, premature rupture of membranes, genital warts, or no antenatal care visits in pregnancy.

Twelve of the 2303 screens were identified as HIV positive within 96 hours of birth, for an overall *in utero* MTCT rate of 0.5%. Eleven of the 12 HIV‐positive infants agreed to participate in the longitudinal EIT Study. All 12 positive infants were among the 369 infants identified as high risk at the time of screening. Only two risk factors were required to identify all 12 positive infants: 9 (75%) were identifiable as high risk by documentation of <8 weeks of maternal ART in pregnancy in the obstetric record, and 3 (25%) by verbal report from the mother that she was not virally suppressed to <400 copies/mL at her last test. This last HIV RNA test was not specified as either in pregnancy, or prior to pregnancy. Additional risk factors identified at the time of screening for the 12 positive infants included poor ART adherence described by one mother (mother of Baby A), and CD4 cell count <250 cells/mm^3^ at last test (mother of Baby E).

Furthermore characteristics of the transmitting mothers are shown in Table 2. It is important to note that each of the transmitting mothers was later confirmed to have detectable HIV RNA at the time of delivery (the HIV RNA for the mother whose infant did not participate in the EIT Study was unknown). Median HIV RNA at enrolment of the transmitting mothers was 9436 copies/mL, and ranged from 67 to 126,093 copies/mL.

**Table 2:** Maternal characteristics for HIV‐positive infants

Infant identifier	Maternal age (years)	Identified as high risk at screening	High‐risk reason	ARVs prior to pregnancy (ever)	ARV regimen during pregnancy	Adherence concerns during pregnancy?^a^	Maternal CD4 (cells/mm^3^)	Maternal VL (copies/mL)
A	28.7	Yes	Less than 8 weeks maternal ART in pregnancy, poor ART adherence in pregnancy.	No	EFV/TDF/FTC^c^	Yes^b^	804	9436
B	20.6	Yes	Detectable maternal viral load at last test	Yes	LPV/r/TDF/FTC	Yes	264	23,912
C	22.8	Yes	Less than 8 weeks maternal ART in pregnancy	Yes	EFV/TDF/FTC	No	336	67
D	21.4	Yes	Detectable maternal viral load at last test	Yes	EFV/TDF/FTC	No	184	125,093
E	29.2	Yes	Less than 8 weeks maternal ART in pregnancy, maternal CD4<250 in pregnancy	No	EFV/TDF/FTC	No	258	355
F	23	Yes	Less than 8 weeks maternal ART in pregnancy	No	None	NA	650	81,982
G	28.5	Yes	Less than 8 weeks maternal ART in pregnancy	No	None	NA	199	25,666
H	32.2	Yes	Detectable maternal viral load at last test	Yes	EFV/TDF/FTC	No	177	54,974
I	30.4	Yes	Less than 8 weeks maternal ART in pregnancy	No	EFV/TDF/FTC	No	791	1389
J	26.5	Yes	Less than 8 weeks maternal ART in pregnancy	No	EFV/TDF/FTC	No	626	2467
K	29.9	Yes	Less than 8 weeks maternal ART in pregnancy	No	None	NA	227	2349
L	27.4	Yes	Less than 8 weeks maternal ART in pregnancy	No	None	NA	Unknown	Unknown
Median	27.95						264	9436

a Identified at enrolment.

b Stopped after 2 weeks (more than 1 month prior to delivery) of ARV due to side effects.

c EFV, efavirenz; TDF, tenofovir; FTC, emtricitabine; LPV/r, lopinavir/ritonavir.

Characteristics of the 12 HIV‐positive infants are shown in Table 3. All infants were screened within 48 hours of birth, at a median age of 19.9 hours (range 6.6 to 44.8 hours). Post‐exposure prophylaxis with ZDV/NVP was dosed at a median of 7 hours after birth (range 0.2 to 115 hours); 9 (75%) infants received prophylactic ZDV/NVP prior to HIV testing. Median CD4 cell count of the infants at enrolment in the EIT Study was 1854 cells/mm^3^ (range 1021 to 5159 cells/mm^3^). Median HIV RNA was 1661 copies/mL (range < 40 copies/mL to >10,000,000 copies/mL). The infant with <40 copies/mL (Baby G) had the presence of virus detected, but it was less than the 40 copies/mL cutoff.

**Table 3:** HIV‐positive infant characteristics

Infant Identifier	Age at first ZDV/NVP dose (Hours)	Age at first positive HIV test (Hours)	Infant CD4 at enrolment (cells/mm^3^)	Infant HIV RNA at enrolment (copies/mL)
A	114.5	18.5	5159	1661
B	25.3	13.6	1995	17,244
C	21.3	25.5	1854	1636
D	7.0	15.9	1021	1,111,950
E	2.4	9.8	1556	1375
F	29.9	6.6	1748	>10,000,000
G	0.2	39.2	1634	<40
H	3.5	19.9	1950	60,247
I	4.2	40.1	1671	3145
J	1.6	44.8	2616	1005
K	23.0	36.5	2177	272
L	—	—	—	—
Median	7.0	19.9	1854	1661

## 4 Discussion

The first year of the EIT Study demonstrated the feasibility of testing for HIV <96 hours from birth for HIV‐exposed infants in Botswana. We determined that all *in utero* MTCT occurred among women with identifiable risk factors at delivery. More than 1900 women whose infants were screened for HIV at birth did not have identifiable risk factors, and there were no *in utero* HIV transmissions among these women. Receiving <8 weeks of ART in pregnancy accounted for 75% of transmissions, and the remaining transmissions occurred among women who knew they were not virally suppressed at the time of their last test. Because this information was easily obtainable at delivery, targeted screening of high‐risk infants is a lower‐cost option than screening the entire birth cohort for the early identification of HIV‐infected infants.

Our data extend the findings of other feasibility studies of birth testing. A recent study in South Africa determined that testing within 48 hours of birth for high‐risk infants was feasible 12. This study identified 11 of 15 HIV‐positive infants as high risk, defining “high risk” as infants born to HIV‐positive mothers who were either first tested positive during labour or had evidence of ART non‐adherence or interruption. Similarly, we identified only one critical risk factor for capturing 75% of transmissions. This finding is important, because it allows health providers to easily identify and target newborns for screening using only the maternal obstetric record, which records ART receipt in pregnancy. In regions where such a record is not available, this is also information that can be determined from a short conversation with the mother. Likewise, a short conversation with mothers was all that was required to identify the remaining 25% of transmissions, as these mothers were aware that they were not virally suppressed at last testing. We did not confirm this actual HIV RNA value, but the infant status was not known at the time of this conversation, and all mothers of positive infants ultimately were found to have detectable HIV RNA at the time of enrolment in the EIT Study.

We were surprised that other risk factors seemed to play little role in determining risk for MTCT, and our initial list of possible risk factors was probably too broad. For example, lack of infant post‐exposure prophylaxis, and lack of maternal ZDV in labour, were not factors needed to determine *in utero* transmission risk (but infant prophylaxis is likely important for intrapartum transmission). Maternal CD4 cell count was reported to be low in only one transmitting mother, though when measured at enrolment median maternal CD4 cell count was only 264 cells/mm^3^. It is not known if there was a drop in CD4 cell count between prior testing and delivery, or whether CD4 cell count was infrequently known at screening, but we suspect the latter. Similarly, though all transmitting mothers were found to have detectable HIV RNA at infant enrolment, maternal HIV RNA was not often available at the time of screening, despite Botswana guidelines during the study period calling for viral load measurements 3 and 6 months post‐initiation of ART and every 6 months thereafter 13. For this reason, the more available risk factor of <8 weeks of ART was a more useful indicator of transmission risk and likely a good proxy for detectable HIV RNA.

Our reported 0·5% *in utero* MTCT rate is lower than most studies and programmes 8, 14, 15, 16. Our MTCT rate may be artificially low because we did not include some infants with potentially higher risk for HIV in our study, including those <35 weeks’ gestational age, <2000 g, with severe medical illness, or those with mothers who were <18 years of age. However, for the most part our study was representative of HIV‐exposed infants in Botswana, and we screened approximately 18% of all HIV‐exposed children in the country during the surveillance 17. Thus, we believe the low MTCT rate accurately reflects the success of the Botswana PMTCT Programme and the extraordinary protection afforded by ART use in pregnancy.

Our study did have some limitations. Within our catchment area, we calculated that our study missed about 44% of HIV‐exposed infants overall. We have no reason to believe that the demographics of those included differed from those who were missed (other than by eligibility criteria we applied). Most of our losses related to the application of eligibility criteria (as highlighted in Figure 1) which would not affect a programme. However, discharge from maternity prior to screening was also an important source of loss, and would be faced by a programme as well. These discharges were often on weekend days when research assistants were not available for screening HIV‐exposed infants; however, with the national implementation of a daily screening process, this number could be significantly reduced. In other regions of Africa, hospital deliveries are uncommon. Additional limitations include the inability to document maternally reported MTCT risk factors, or to quantify the number of women for whom risk factors were unknown. The ability to assess potential risk factors (especially HIV RNA or CD4 cell count) will vary across settings. However, our study provided a good assessment of what was known at typical delivery sites in Botswana, and importantly, the duration of ART should be universally available.

The earliest possible identification of HIV infection has several important benefits for HIV‐infected neonates. First, it enables early initiation of ART in infants, which leads to better clinical and immunological outcomes than deferred ART 2, 3. Second, studies of children who initiated early ART demonstrate low levels of markers of HIV persistence as well as undetectable HIV‐specific immune responses 3, 5, 18. These findings suggest the potential for better long‐term outcomes among early‐treated infants 19. An important consideration for any programme that considers birth testing – whether targeted or universal – is that birth testing only identifies *in utero* transmissions. Therefore, birth testing can complement and improve existing programmes that rely on 6 to 10 week testing, but does not replace later testing.

## 5 Conclusions

In conclusion, our study identifies maternal characteristics that help target birth testing of high‐risk infants. This practice can identify the large majority of *in utero* HIV transmissions, and allows early ART initiation for these infants. Birth testing has been shown to be feasible, acceptable and affordable 20, but due to limited resources a targeted approach may be the best initial method to implement this important intervention.

## Competing interest

The authors declare that they have no competing interests.
